# Severe acute respiratory syndrome coronavirus 2 transaminase elevation likely of non‐hepatic origin, with protection from older age and vaccination

**DOI:** 10.1002/jpr3.70034

**Published:** 2025-05-20

**Authors:** Antonia F. Ovale, Cassandra Charles, Janet Rosenbaum, Priscila Villalba‐Davila, Shagun Sharma, Saema Khandakar, Thomas Wallach

**Affiliations:** ^1^ Department of Pediatrics SUNY Downstate Health Sciences University Brooklyn New York USA; ^2^ SUNY Downstate Health Sciences University Research Foundation Brooklyn New York USA; ^3^ Department of Biostatistics and Epidemiology SUNY Downstate Health Sciences University, School of Public Health Brooklyn New York USA; ^4^ Department of Pediatrics Division of Pediatric Gastroenterology SUNY Downstate Health Sciences University Brooklyn New York USA; ^5^ Department of Pediatrics Kings County Medical Center Brooklyn New York USA

**Keywords:** acute infection, COVID, hepatitis

## Abstract

Severe acute respiratory syndrome coronavirus 2 (SARS‑CoV‑2) has known liver tropism. Multiple reports and studies demonstrated liver injury early in the pandemic. This retrospective cross‐sectional comparison evaluates predictors of transaminase elevation during acute SARS‐CoV2 infection, with particular interest in evaluating whether vaccination is associated with transaminase elevations. We extracted electronic medical record data for pediatric SARS‐CoV2 patients presenting at safety net hospitals in Brooklyn, NY, between March 2020 and March 2022 with a coincident comprehensive metabolic panel, without multisystem inflammatory syndrome in children, prior liver disease and sickle cell disease (*n* = 133): 79.2% Black and 87% non‐Hispanic. Transaminase elevation was more common among younger patients and patients requiring hospitalization or intensive care unit care. Vaccination was associated with lower quantitative levels of transaminase elevation but not the binary indicator for elevated transaminases. In aggregate, our results suggest transaminase elevation is a function of SARS‐CoV2 disease severity.

## Abstract (highlights)

**What is Known:**

- Past research suggests severe acute respiratory syndrome coronavirus 2 (SARS‑CoV‑2) causes liver injury and potentially liver failure.
- SARS‐CoV2 infection has had variations in severity and involved tissue types between variants and with expanding population immunity.

**What is New:**

- Vaccinated and older pediatric patients have lower risks of transaminase elevation, but age and vaccination could not be decoupled due to age‐specific vaccine eligibility.
- Association between transaminase elevation and level of care, unvaccinated status, and younger age suggests it may be a function of disease severity.

## 1 INTRODUCTION

It has been reported that severe acute respiratory syndrome coronavirus 2 (SARS‑CoV‑2) infections can trigger elevations to serum transaminases, raising concerns for direct liver injury.^1^ It is not clear how acute viral infection impacts the pediatric liver, nor what changes have occurred with evolved variants and acquired population immunity.^2^ In the adult population, it has been reported that approximately 20%–30% of patients who were hospitalized due to SARS‐CoV2 acute infection presented with abnormal liver laboratory tests, characteristically presenting with mild cholestasis and elevation of transaminases.^3^ Mechanistic work has suggested that direct viral injury to the liver is plausible.^4^ Pediatric case series studies have suggested the viruses have the potential to drive liver failure^5^ yet further understanding of the natural history of this disease in pediatrics is needed, especially as meaningful interventions such as vaccination have been deployed.

The virulence of SARS‐CoV2 infection has changed over time with increased population immunity due to vaccination and past infections,^6^ and pediatric hepatic sequelae of SARs‐CoV2 infection may have changed from previous studies.^7^, ^8^ In this study, we assess the natural history, population immunity, and associated risks of acute laboratory‐confirmed SARS‐CoV2 infection on the liver at neighboring safety‐net hospitals in Brooklyn, NY between March 2020 and March 2022. We hypothesized that transaminase elevations would be associated with increased body mass index (BMI), a higher level of treatment care, and more prevalent in the early phases of the epidemic. We anticipated milder symptomatology and lower rates of transaminase elevation among children who had prior immune exposure from infection or vaccination.

## 2 METHODS

### 2.1 Ethics statement

Approval for a retrospective chart review was granted by the SUNY Downstate Institutional Review Board.

### 2.2 Data

We tested our hypotheses using data from 133 patients ages 0–18 years (median age 10 years, interquartile range 2–15 years), among whom 79% were Black and 87% non‐Hispanic. We conducted a retrospective review of electronic medical records of pediatric patients evaluated for SARS‐CoV2 between March 2020 and March 2022 at two safety net hospitals in Brooklyn, including one with the highest Medicaid percentage in the state.^9^ Patients were evaluated at the emergency departments (ED) before being admitted to the pediatrics floor or pediatric intensive care unit (PICU). No patients were repeated. We included patients ages 0–18 years with an ED or hospital visit to either institution where they received a diagnosis of SARS‐CoV2 infection via rapid antigen test or polymerase chain reaction and had a comprehensive metabolic panel drawn during that clinical encounter up to 2 days after the SARS‐CoV2 infection diagnosis. We excluded patients with a diagnosis of multisystem inflammatory syndrome in children, prior history of transaminase elevation or liver disease, and history of sickle cell disease, due to confounding effects on transaminases. We reviewed 3452 patient charts from 03/01/2020 to 03/01/2023, among whom 133 patients met inclusion criteria.

### 2.3 Measures

Our primary outcomes were aspartate aminotransferase (AST) and alanine aminotransferase (ALT) elevation, which were measured both quantitatively and with a binary indicator using cutoffs from Bussler et al. (AST > 46, ALT > 20).^10^

Our primary exposure variable was immunity, based on vaccination and prior infection status, vaccination status was obtained from the New York Citywide Immunization Registry. To assess vaccination and prior infection, we obtained data on documented SARS‐CoV2 Spike IgG antibody formation; we included these data in the analysis if antibodies were detected before or at the onset of acute SARS‐CoV2 illness. Vaccination status is highly endogenous to age and date of presentation because the US Food and Drug Administration emergency use authorization for the vaccine approvals by age groups: the vaccine was authorized for ages 12 and older on May 10, 2021; for ages 5–11 on October 29, 2021; and ages 0–4 in June 2022. In our sample, only three patients ages 5–11 were vaccinated.

### 2.4 Other covariates

Demographic measures included age in months, male versus female sex assigned at birth, Black versus nonblack race, and Hispanic versus non‐Hispanic ethnicity. We created age groups according to vaccination eligibility: ages 0–4, ages 5–11, and ages 12–18 years.

We assessed the following gastrointestinal (GI) symptoms at presentation: nausea, vomiting, diarrhea, and decreased oral intake. We also created a binary indicator for the presence of any GI symptom.

We defined the following weight categories: normal or underweight as BMI percentile 0‐84, overweight as BMI percentile 85–94, and obesity as BMI percentile 95–100. When only BMI was available instead of BMI percentile, we converted BMI to BMI z‐scores using US Centers for Disease Control growth charts (https://www.cdc.gov/growthcharts/percentile_data_files.htm).

Level of care had the following values: admitted to pediatrics floor, admitted to PICU, or discharged from the ED after initial encounter with no follow‐up.

We created a time indicator with the following categories that correspond with vaccine access: March to December 2020 (none), January to June 2021 (ages 16–18 eligible for vaccination), June to November 2021 (age 12–18 were eligible for vaccination), and November 2021 to March 2022 (ages 5–18 years were eligible for vaccination).

### 2.5 Analysis

We evaluated the association between elevated transaminases and continuous variables (age in months, AST/ALT ratio, BMI *z*‐score) using Kolgomorov–Smirnov to evaluate the difference between the areas between the curves. We evaluated the association between elevated transaminases and binary variables using the non‐parametric Wilcoxon test, and with multicategory variables using the chi‐square test with Holm‐adjustment for pairwise comparisons. We produced plots using ggplot2.^11^ Elevated transaminases were present in 53/133 patients; because the outcome was common, we used multivariate Poisson regression. We used causal mediation analysis to quantify how much of the association between vaccination and elevated transaminases is mediated by age, using the mediation package.^12^ All analyses used R version 4.4.2.

The SUNY Downstate institutional review board and Kings County STAR committee approved this study (approval # 1888245‐1).

## 3 RESULTS

**Figure 1:**
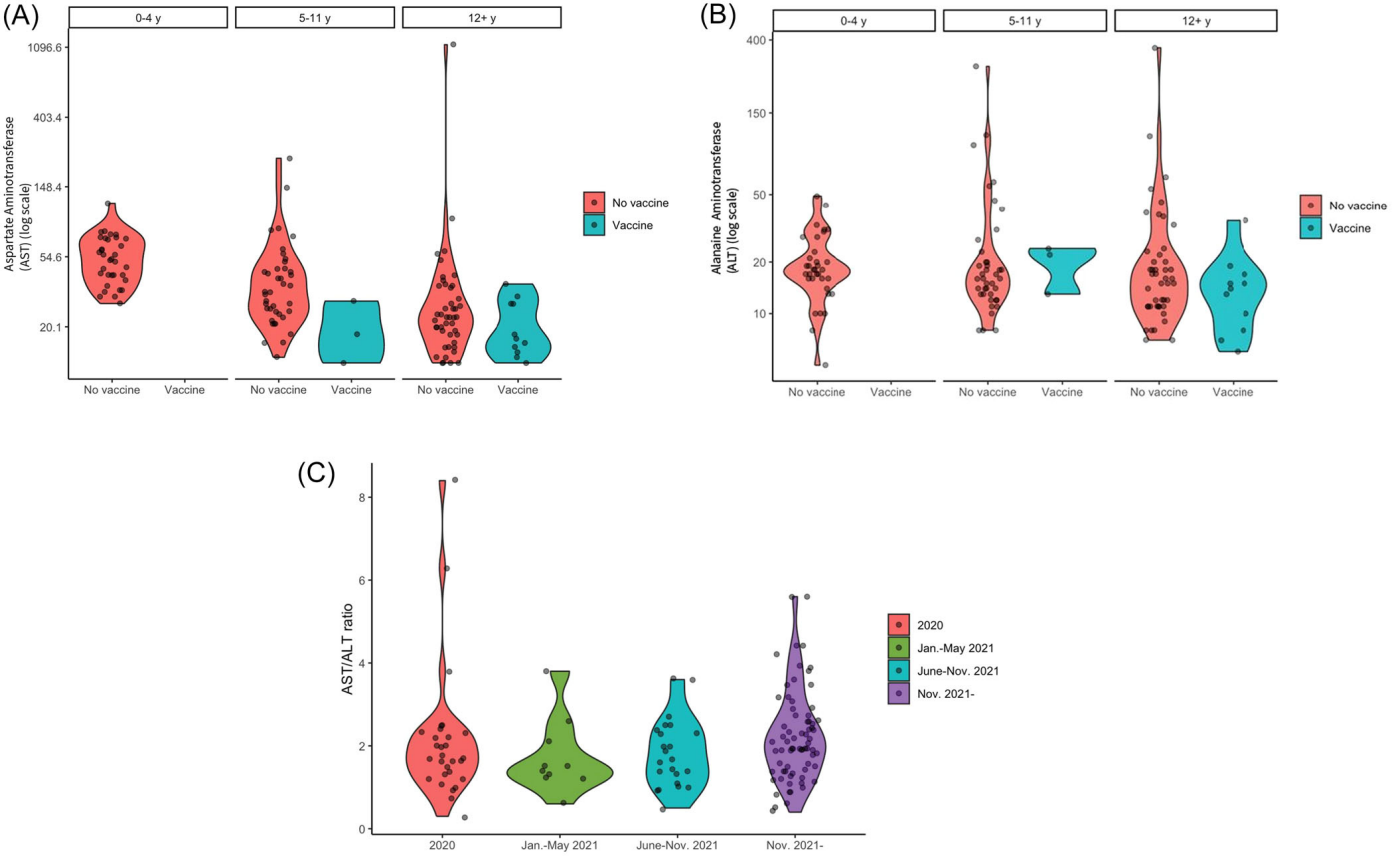
(A) Violin plot of AST and (B) violin plot of ALT elevation by vaccination status. (C) AST/ALT ratio by variant window. ALT, alanine aminotransferase; AST, aspartate aminotransferase.

Among these pediatric patients presenting in the safety net hospital EDs with SARS‐CoV2, 10.5% were vaccinated and 39.8% had elevated transaminases (Table 1). All patients had normal values of international normalized ratio, and further chart review did not demonstrate any patients with progressive liver injury or evidence of liver failure or complications of liver injury. Vaccinated children had lower AST (median 17.5 interquartile range [IQR] [14.2, 28]) than unvaccinated children (median 34.0 and IQR [23.0, 52]) (Figure 1). AST levels in unvaccinated patients were significantly higher (34.0 vs. 17.5, *p* < 0.001).

**Table 1:** Demographics and biomarkers at time of presentation by vaccination status.

	No vaccine (*n* = 119)	Vaccine (*n* = 14)	*p*‐Value
Transaminemia	50 (42.0%)	3 (21.4%)	0.2
AST, median, IQR	34.0 (23.0, 52)	17.5 (14.2, 28)	<0.001
AST elevations			0.2
None (normal AST ≤ 46)	85 (71.4)	14 (100)	
Mild (abnormal, less than 2x ULN)	29 (24.4)	0	
Moderate (between 2‐5x ULN)	4 (3.4)	0	
Severe (more than 5x ULN)	1 (0.8)	0	
AST/ALT ratio, median, IQR	1.90 (1.36, 2.54)	1.40 (1.06, 1.85)	0.02
ALT, median, IQR	17.0 (12.0, 22.5)	14.5 (10.8, 18.5)	0.2
ALT elevations			0.8
None (normal ALT ≤ 20)	87 (73.1)	11 (78.6)	
Mild	18 (15.1)	3 (21.4)	
Moderate	10 (8.4)	0	
Severe	4 (3.4)	0	
Random glucose, median, IQR	97.0 (89.0, 107.0)	96.0 (88.0, 121.5)	0.9
Albumin, median, IQR	4.50 (4.1, 4.8)	4.35 (4.1, 4.8)	0.8
Alkaline phosphatase, median, IQR	208 (113, 258)	117.5 (60.8, 220)	0.1
Platelets, median, IQR	267 (207, 368)	298.5 (235.8, 362.3)	0.5
Total bilirubin, median, IQR	0.40 (0.2, 0.6)	0.40 (0.2, 0.6)	0.8
Age (months), median (IQR) (range)	108 (26, 180)	186 (156, 204)	0.001
Age group			0.007
0–4 years	34 (28.6%)	0	
5–11 years	40 (33.6%)	3 (21.4%)	
12–19 years	45 (37.8%)	11 (78.6%)	
Hospital			0.03
Downstate	32 (26.9%)	8 (57.1%)	
Kings County	87 (73.1%)	6 (42.9%)	
Date presenting			0.003
March–December 2020	29 (24.4%)	0	
January–June 2021	10 (8.4%)	0	
June–November 2021	23 (19.3%)	0	
November 2021–March 2022	57 (47.9%)	14 (100%)	
Black race	90 (78.3%)	12 (85.7%)	0.7
Non‐Hispanic ethnicity	105 (91.3%)	13 (92.9%)	1.0
Male sex	63 (52.9%)	9 (64.3%)	0.4
Pre‐existing conditions	52 (43.7%)	11 (78.6%)	0.01
Asthma	16 (13.4)	6 (42.9)	0.01
BMI, median, IQR	19.2 (16.8, 24.5)	25.6 (19.0, 27.0)	0.02
BMI *z*‐score, median (IQR)	0.47 (–0.40, 1.67)	1.21 (0.24, 1.81)	0.2
BMI *z*‐score, mean (SD)	0.41 (1.75)	1.02 (1.25)	0.1
BMI class			0.2
Normal weight or underweight	68 (62.4%)	6 (42.9%)	
Overweight (>85th percentile)	20 (18.3%)	3 (21.4%)	
Obese (>95th percentile)	21 (19.3%)	5 (35.7%)	
Missing	10 (8.4%)	0	
Presenting with GI symptoms	63 (52.9)	10 (71.4)	0.2
Presenting with nausea	31 (26.1)	3 (21.4)	1.0
Presenting with diarrhea	14 (11.8)	2 (14.3)	0.7
Presenting with decreased intake	18 (15.1)	1 (7.1)	0.7
Presenting with abdominal pain	22 (18.5)	5 (35.7)	0.2
Presenting with other GI symptoms	22 (18.5)	4 (28.6)	0.5
Level of care			0.3
ED and discharged	56 (47.1)	10 (71.4)	
Floor	45 (37.8)	3 (21.4)	
PICU	18 (15.1)	1 (7.1)	

*Note*: *p*‐Value for continuous variables was from Wilcoxon test due to skewed distributions. *p*‐Value from categorical variable was from chi‐square test.

Abbreviations: ALT, alanine aminotransferase; AST, aspartate aminotransferase; BMI, body mass index; ED, emergency departments; GI, gastrointestinal; IQR, Interquartile range; PICU, pediatric intensive care unit; SD, standard deviation; ULN, upper limit of normal.

Younger children were more likely to have elevated transaminases, which had prevalence of 61% among patients ages 0–4, 41% among patients ages 5–11, and 25% among patients ages 12–18 (*p* = 0.0001). Patients with normal transaminase levels were on average 784 months older than patients with elevated transaminase (median 144 [69, 201] months vs. 60 [13, 144], *p* < 0.001) (Table 1). Each year of age was associated with 7% fewer cases of transaminase elevation (prevalence ratio = 0.93, 95% confidence interval [CI] [0.89, 0.97]) (single variable Poisson regression not shown). Patients older than 12 years old had 62% lower risk of elevated transaminases than ages 0–11 years old (Table 1).

In causal mediation analysis, 51% of the effect of the transaminase elevation in vaccinated individuals was mediated by a binary indicator for being age 12 or older, controlling for pre‐existing condition and presenting during omicron (*p* = 0.04), and we were unable to determine a clearly significant protective effect of vaccination for incidence of transaminase elevation. In a second analysis, 69% of the shift in transaminase elevation frequency in vaccinated patients is mediated by age in months, controlling for pre‐existing condition and presenting during omicron (*p* = 0.02).

Rates of elevated transaminases appeared to vary with time. A greater proportion of patients with higher transaminase elevation required admission to the hospital and/or the PICU, compared with patients discharged from ED (Table 1). Fifty‐six percent of patients presented with one or more GI symptoms. 47.2% patients with elevated transaminases presented with one or more GI symptom at initial encounter. There was no association between the presence of GI symptoms and transaminase elevation status (*p* = 0.20). There was also no association between vaccinated and nonvaccinated patients and the presence of GI symptoms at onset (*p* = 0.2). Interestingly, there was no difference in BMI between patients that had elevated transaminases and the patients that did not (*p* = 0.2).

## 4 DISCUSSION

Our study is among the first reports to assess SARS‐CoV2 associated transaminase elevation by vaccine status, over variant windows, and in a primarily minoritized population. A focus on a minoritized and low‐income population is highly relevant, as this population remains most at risk of SARS‐CoV2 infection.^13^ We note a significant age association (higher risk in younger children) and a shift in characteristic of transaminase elevation over time. We hypothesize that this suggests that currently AST elevation associated with SARS‐CoV2 infection is driven primarily by disease severity. However, we do note a higher median ALT in the impacted cohort, suggesting that multi‐system involvement may still be generating hepatic injury. Notably, vaccination, an intervention known to reduce disease severity, decreases ALT elevation significantly, supporting the interpretation that the more directly hepatotropic effects of SARS‐CoV2 appear to be waning.

In aggregate, these findings create a picture reflective of transaminase elevation as a process of disease severity, although it remains possible that immunogenicity has also shifted with variants. AST is a nonspecific enzyme which can derive from multiple tissues, including muscle and red blood cells. The shifting De Ritis ratio over time and the normal ALT levels reported here suggest that increasingly transaminase elevations associated with SARS‐CoV2 infection are driven by nonhepatic sources.^14^ The ratio represents the time course and aggressiveness of disease that would be predicted from the relatively short half‐life of AST (18 h) compared to ALT (36 h), and can be used to infer origin of transaminase elevation.^14^ As the AST/ALT ratio increased over the time (Figure 1), in the context of the high frequency of AST elevation with normal range (if elevated over the normal transaminase group) ALT levels, it is highly likely that transaminase elevation has increasingly become a reflection of nonhepatic tissue injury.

This finding is consistent with our findings in several ways. Our results concur with the prior findings that pediatric patients with elevated transaminases were more likely to require higher level of care. Our finding of a strong age linkage is also consistent with this as more severe disease has been described in younger children with SARS‐CoV2 infection.^15^ AST was significantly lower in vaccinated patients, suggesting a protective impact, although the median levels of both vaccinated and nonvaccinated patients were within the normal range; this association with age is possibly a function of age‐based vaccine access, and we hypothesize that a strong vaccine protection would be noted in a larger study. We propose that this vaccine effect is likely to be driven by reduced illness severity because SARS‐CoV2 vaccination provides a strong protective effect against severe illness.^16^

Interestingly, BMI elevation was not associated with transaminase elevation in this study. A higher BMI is thought to be a risk factor for SARS‐CoV2 severity and transaminase elevation secondary to nonalcoholic steatohepatitis or nonalcoholic fatty liver disease (NAFLD),^17^, ^18^ which may be due to the study's demographic composition because NAFLD is uncommon in patients of African descent.^19^, ^20^

We had theorized that GI symptoms would correlate with transaminase elevation if SARS‐CoV2 directly caused hepatocellular injury, due to blood flow from the GI tract to the liver, but we found no association in our data (Table 1), which again is consistent with a reduced direct hepatotropic effect.

Our study had several limitations. Our study sample was not representative of all SARS‐CoV2 infections presenting in the hospital EDs at this time because frequently mild infections do not result in obtaining blood tests such as a comprehensive metabolic profile; however, we expect that the inclusion criteria have not biased the association. This study was also limited by the inability to trend results over time, limited demographic composition (overwhelmingly black) and small sample size.

## 5 CONCLUSION

This retrospective cross‐sectional study of SARS‐CoV2 associated transaminase elevation has identified major risk factors of young age and disease severity, and highly suggests a strong protective effect from vaccination. It also suggests that while liver injury may play a role, especially in severe cases, the predominant mechanism driving transaminase elevation is likely nonhepatic AST elevation, although further study is needed.

## CONFLICT OF INTEREST STATEMENT

The authors declare no conflict of interest.
